# A Review of Cordocentesis: Percutaneous Umbilical Cord Blood Sampling

**DOI:** 10.7759/cureus.16423

**Published:** 2021-07-16

**Authors:** Nikhil Chowdary Peddi, Chaithanya Avanthika, Sravya Vuppalapati, Ramya Balasubramanian, Jaskaranpreet Kaur, Chaithanya Datta N

**Affiliations:** 1 Pediatrics, People's Education Society Institute of Medical Sciences and Research, Kuppam, IND; 2 Pediatrics, Karnataka Institute of Medical Sciences, Hubli, IND; 3 Pediatrics, Smt. Kashibai Navale Medical College, Pune, IND; 4 Otolaryngology, Dayanand Medical College, Ludhiana, IND; 5 Pediatrics, Jawaharlal Nehru Medical College, Belgaum, IND

**Keywords:** fetal anemia, maternal and fetal outcome, pubs, cordocentesis, fetal monitoring

## Abstract

We discuss the current indications, technical variation and procedure-related complications of percutaneous umbilical cord blood sampling (PUBS). The term PUBS is commonly used in the United States. Cordocentesis and funipuncture are equivalent terms. A needle guided by ultrasound is introduced into a blood vessel (usually the vein) of the umbilical cord to collect fetal specimen in PUBS.

We conducted a literature search in PubMed indexed journals and analyzed all related articles on PUBS and cordocentesis. We chose this subject because it is a relatively new but convenient method that has both diagnostic and therapeutic value in fetal medicine. At present the only procedure that provides direct access to fetal circulation is PUBS. The most common clinical indication for PUBS is suspected fetal anemia. Other major indications for PUBS are the diagnosis of congenital infections, cytogenetic analysis, metabolic disorders, fetal growth restriction and hematologic disorders. Therapeutic applications of cordocentesis or puncture of the umbilical cord are in utero transfusions for rhesus alloimmunization and medication administration. PUBS also provides a direct assessment of fetal thyroid function diagnosing fetal thyroid disorders and helps administer therapy in utero.

Literature demonstrates a low incidence of complications associated with percutaneous umbilical blood sampling. For PUBS, the true complication rate related to the method of sampling remains unclear. A few cases reported complications conducted PUBS for therapeutic purposes which naturally has a higher accident rate compared to diagnostic purposes. Although life-threatening complications are rare, there are potential risks that include bleeding from the puncture site, fetal bradycardia, vertical transmission of maternal infection. Therefore, PUBS should be performed at perinatal care centers by experienced physicians and the best time is between 17 to 40 weeks of gestation. There are three methods used to approach the umbilical cord that includes direct, indirect and free puncture. Anteriorly placed placenta allows an easier approach to the umbilical cord. The danger of abruption of placenta must be kept in mind while using this technique. The number of punctures should be limited to a maximum of 3 to reduce complications. According to a case series report, the mean time required for the procedure was 4 minutes with a fall in duration seen with increased experience.

In conclusion, percutaneous blood sampling allows direct access to fetal circulation thus opening up new areas of prenatal diagnosis and therapy. PUBS is now a well-codified procedure. It is clear from our literature review that risks directly related to the technique are small. The indication of the procedure must be carefully chosen as the risk of complications of umbilical cord puncture is directly related to the severity of the condition. Complications such as bleeding and hematoma formation are related to duration and number of punctures which are operator-dependent. Thus, only highly trained personnel should conduct the procedure. The list of indications is extensive and growing. Nevertheless, this technique shows potential to open up new realms in the area of fetal medicine.

## Introduction and background

Pathological biomarkers of the fetus are routinely collected via percutaneous umbilical cord blood sampling (PUBS). This advanced technique is safe and convenient and can be performed in the second and third trimester of pregnancy by direct puncture of the umbilical vein close to its placental insertion, by employing thin needles which are guided using ultrasound [1]. Apart from fetal sampling, PUBS also finds its use in intra-uterine transfusion and administering medications to the fetus [2]. The incidence of procedure-related complications, including bleeding from the umbilical cord puncture site and an abnormal fetal heart rate, ranges from 5% to 30% [2,3] and hence are considered rare. The advantages of this procedure outweigh the risks.

Percutaneous umbilical cord blood collection should be done by experienced physicians in prenatal care centres; however, there has been no comprehensive study of the procedure's clinical characteristics, yet. The principal clinical indication for PUBS is the diagnosis of fetal anemia owing to red-cell allo-immune diseases around the world. The key applications of this procedure are diagnosis and identification of fetal infections, karyotype analysis, diagnosis of hematologic conditions, fetal growth retardation, and metabolic analysis. This procedure is becoming more popular in recent times, since it provides direct data on fetal blood status [4,5]. The purpose of this study is to clarify and review current practices regarding PUBS, including its indications, uses, and procedure-related complications.

## Review

Alloimmunization in pregnancy

Serial analysis of the unanticipated antibody titer in the mother's serum by the indirect antiglobulin examination is the conventional primary step in the therapy of people in jeopardy for alloimmune hemolytic condition. If the mother's antibody titer surpasses the usual limit which is 8, or if there is a previous obstetric background of alloimmunization, additional screening for fetal hemolytic condition is advised [6].

Percutaneous umbilical blood testing is an efficient brand-new tool for establishing whether a fetus is at threat of developing isoimmune hemolytic anemia [6]. This approach enables direct hematocrit as well as blood group analyses, along with a direct antiglobulin analysis of the fetus [6]. If the fetus is discovered to be antigen-negative for the mother's antibody of issue, additional therapies, such as numerous amniocentesis, can be prevented [6]. Bell and Weiner study discovered 23% of fetuses to be antigen-negative in an example of 128 successive individuals evaluated for alloimmunization, without any requirement for extra intrusive treatments [7].

Using PUBS is not simply restricted to medical diagnosis, however, can additionally be utilized for restorative intervention. Percutaneous Intravascular transfusion is carried out utilizing the very same technique as testing. When a fetal blood sample is taken as well as if the fetus is found to be anemic, the needle is kept in the lumen of the umbilical cord vessel and packed red cells are transfused right into the fetus [6]. The fetal hemoglobin and hematocrit are determined after transfusion and additional blood is provided as required [6].

Direct intravascular transfusion has actually minimized the requirement for preterm delivery, enabled earlier treatment than formerly feasible with intraperitoneal transfusion (IPT), enhanced hydrops survival, and boosted general perinatal end result in isoimmune hemolysis fetuses [7].

In cases of uncommon antibody detection, such as anti-Kell, PUBS has actually enhanced medical diagnosis accuracy. Too and Berkowitz determined a case of intrauterine death triggered by Kell sensitization in which amniotic fluid evaluation disclosed no delta OD450 elevations [9]. Inspite of doing several intraperitoneal transfusions for some fetuses which were anticipated to be severely ill, Kell negative baby was born. Therefore, direct fetal blood sampling for antigenicity as well as anemia monitoring could be much more reliable for medical diagnosis of Kell alloimmunization [7].

Fetal karyotyping

In contrast to an amniotic fluid cell culture, cytogenetic research study of fetal lymphocytes collected by percutaneous umbilical blood sampling can be finished in two to three days [6]. This time delay might be necessary if a pregnancy termination decision is immediate, such as when the legal abortion time frame approaches. Rapid karyotyping commonly lowers parental stress and anxiety triggered by a long haul for outcomes [6]. Mosaicism if observed on amniotic fluid cell culture or chorionic villus sampling might be confirmed utilizing fetal blood sampling [6].

Congenital infections

It is very important to collect fetal blood at a time when the fetus is capable of mounting a considerable immune reaction to an antigenic stimulation [6]. Prenatal medical diagnosis of rubella often tends to be more reliable in fetal blood samples by identifying the IgM antibodies in the 22nd week of pregnancy [6,8]. Virus detection in chorionic villi samples or amniotic fluid samples are two options [8]. Nevertheless, these approaches for identifying rubella infection in amniotic fluid and chorionic villi might be unreliable, specifically amniotic fluid because of reduced viral load in amniotic fluid samples [8]. According to some researches, rubella virus can be found in the placenta but not in the fetus, or it can be found in the fetus but not in the placenta, leading to false-negative findings.

Cytomegalovirus (CMV) is detected by identifying IgM antibodies in fetal blood at 25 weeks of pregnancy [6].In the medical diagnosis of CMV infection, PUBS is utilized along with amniocentesis as well as ultrasound [7]. Antiviral agents such as ganciclovir can currently be used in the management of CMV in infants; however, in utero therapy is not available. Therefore, the present function of PUBS in CMV disease is diagnostic rather than therapeutic [7]. Varicella can be detected in fetal blood by cordocentesis at 32' 1/2 weeks of gestational age [6]. 

In congenital toxoplasmosis, antibody synthesis is usually delayed. Generally, it is possible that affected babies do not begin producing antibodies till, several months after birth. Toxoplasma infection is identified prenatally by serologic tests, fetal blood as well as amniotic fluid cultures, hematologic as well as hepatic enzyme analyses, and ultrasound assessments [6].

Infection with the human parvovirus B19 during pregnancy has been connected to a greater risk of fetal death [7]. Parvovirus B 19 infection is associated with aplastic anemia in patients with hemolytic disorders such as sickle cell anemia as well as hereditary spherocytosis. It is frequently related to erythema infectiosum, also called the fifth disease, which presents with characteristic skin involvement in kids. PUBS is a reliable method for determining as well as treating hydropic fetus affected with the B19 virus in utero. Fetal gamma immunoglobulin M (IgM) titers are measured on cord blood samples to confirm acute infection and several case reports have actually shown the possibility to reverse hydrops [7]. Prenatal screening for congenital HIV infection in amniotic fluid or cord blood is not advised due to the fact that the procedure increases the risk of the transfer of infection from the mother to the fetus [8].

Intra uterine growth restriction (IUGR)

The function of PUBS in the diagnosis of congenital infections as well as chromosomal irregularities, two important etiologies of IUGR, was explained formerly. The technique can also be utilized to collect fetal blood for biochemical evaluation, enabling a more accurate assessment of fetal health and wellness that can affect obstetrical choices [6].

Reduced pH as well as oxygen saturation, along with greater pC02 and also lactate levels, are discovered in IUGR. A pH less than 7.30 in umbilical vein and a lactate concentration greater than 1.5 mmol have actually been reported as bad prognostic signs [6].

Using PUBS to detect fetal hypoxia and acidosis is a considerable innovation in the analysis of placental insufficiency, fetal growth retardation, as well as fetal distress. Cordocentesis is additionally utilized to identify the effect of long-term maternal hyperoxygenation on the fetal PO2 of growth-retarded fetuses.

Fetal thrombocytopenia

Percutaneous umbilical blood sampling might be utilized to identify the fetus platelet phenotype as well as assess the level of alloimmune thrombocytopenia as early as 20 weeks of pregnancy [6]. In addition, the treatment allows in utero platelet transfusions, which can minimize the risk of intracranial bleeding [6]. At 20 to 22 weeks of pregnancy, all patients at risk for neonatal alloimmune thrombocytopenia need to undertake cordocentesis [6]. Numerous approaches have actually been recommended if the fetus is positive for PIA1 and is already thrombocytopenic. Repeated in utero platelet transfusions were recommended by some [6].

Others have actually rejected this option as impractical, pointing out the half-life of platelets, which requires at least once a week transfusions, with the risks of several intrusive procedures and also transfusion-transmitted infection [6]. One more alternative is to recommend patients to avoid injury and also to have serial ultrasound scans with specific interest to the fetal mind. At about 37 weeks of pregnancy, when fetal lung maturation is anticipated, a second fetal blood sample is taken, and if the fetus is thrombocytopenic, concentrated maternal platelets are transfused and a repeat platelet count is done at the end of the treatment. This strategy does not remove the possibility of fetal blood loss during pregnancy [6].

Although fetal thrombocytopenia can take place as early as 20 weeks of pregnancy, just two cases of intracranial hemorrhage have actually been recognized prior to the 30th week. As a result of this finding, a "compromise strategy" has actually been suggested, in which weekly platelet transfusions are begun in between 26 and 30 weeks of pregnancy and proceeded till fetal maturation enables delivery [6]. Transfusions are limited to the time when there is the greatest risk of fetal hemorrhage [6].

Idiopathic thrombocytopenic purpura is the production of maternal autoantibodies against platelets triggers thrombolysis in the reticuloendothelial system, particularly in the spleen [7]. Mother's immunoglobulin G can cross the placenta and cause fetal platelet phagocytosis.

Genetic disorders

Percutaneous umbilical blood testing can be made use to identify a range of congenital diseases while pregnancy [6]. Most of these conditions can be detected by making use of molecular DNA analysis of fetal cells accumulated via amniocentesis or chorionic villus testing [6]. Nevertheless PUBS works, when a blood sample is needed for medical diagnosis, when a person fails to reveal early in pregnancy and also requires a fast medical diagnosis, or when other techniques have actually fallen short to offer a conclusive outcome [6]. The investigation of fetal blood loss issues has actually been simplified, thanks to percutaneous umbilical blood testing. Coagulation factors in fetal blood might be utilized to detect hemophilia [6]. The clumping of ristocetin in unborn children in danger for von Willebrand's condition is measured. Factor VIII coagulant activity as well as von Willebrand's factor antigen are likewise determined [6].

PUBS, as formerly specified, has a tendency to be exceptionally risk-free, also when it comes to fetal hemorrhagic disorders. Two babies with Glanzmann's thrombasthenia, one with severe von Willebrand's condition, as well as one with hemophilia have actually been reported with extreme blood loss until now [6]. Fetal blood testing can additionally be made use for medical diagnosis of immunodeficiency disorders by gauging the enzymes adenosine deaminase and also purine nucleoside phosphorylase [6].

Thyroid disorder

Stillbirths, intrauterine development retardation, cardiac arrest, as well as postnatal developing problems have actually all been connected to fetal hyperthyroidism, while hypothyroid infants might display mental retardation, in addition to motor and learning impairments [7]. PUBS offers straight accessibility to fetal thyroid illness medical diagnosis as well as the capacity to direct the management of treatments in utero [7].

Bell and Weiner study recognized the therapy of fetal goitre triggered by believed fetal hypothyroidism in a mom that had actually been recommended propylthiouracil for Graves' disease. The intraamniotic use of thyroxine led to significant decrease in fetal goitre size as well as routine neonatal thyroid examinations [7]. The benefit of PUBS is that it permits direct analysis of fetal thyroid function as well as therapy modulation [7].

Fetal tachyarrhythmias

Fetal tachyarrhythmias, such as supraventricular tachycardia and also atrial flutter/fibrillation, might trigger heart disease, along with nonimmune hydrops [7]. Several writers have actually reported success in dealing with fetal tachyarrhythmias in non-hydropic unborn children via transplacental passage of antiarrhythmic medications such as digoxin, verapamil, amiodarone, and also others after mother's treatment [7].

Nevertheless, as a result of placental disorder, transplacental treatment is much less efficient in hydrops fetuses and also has actually been connected to inadequate fetal end results [7].In these instances, direct fetal intravascular treatment provides a method to deal with these seriously harmed unborn children [7].

Complications of PUBS

Infection, preterm premature rupture of membranes, premature birth, abruption of the placenta, hemorrhage, alloimmunization, bradycardia in the fetus and pregnancy loss are all hazards associated with this procedure [9].

Fetal bradycardia occurs in 5% of patients during or shortly after the procedure, with the rate decreasing with increasing fetal age. This can happen as a result of vasospasm of umbilical artery. Streaming from the needle insertion site into the fetal artery happens in 20% to 30% of cases and is usually temporary, but it can occasionally lead to fetal exsanguination. Cord compression and fetal bradycardia may result from an iatrogenic umbilical cord hematoma. This is usually asymptomatic or temporary, but if there is continuous evidence of unsettling fetal condition, such as prolonged fetal bleeding, it may necessitate delivery. One study found a 2.4% emergency caesarean delivery rate, which was linked to birth hypoxia in 73% of cases and newborn death in 33%. In another study, 5% of individuals with alloimmune thrombocytopenia (NAIT) who had diagnostic cordocentesis had an emergency caesarean delivery. According to some sources, fetal loss rates after cordocentesis range from 1.4% to 1.9% [9].

Most diseases can now be diagnosed using chromosomal or genetic tests rather than histological or enzymatic diagnostics, thanks to advances in diagnostic procedures that have incorporated genetics. Due to the ability to diagnose fetal anemia and obtain a quick chromosomal analysis, cordocentesis is also used in some circumstances.

(a) Transplacental puncture of the cord attachment site- If the placenta is attached along the anterior uterine wall, transplacental puncture of the vena umbilicalis could be performed near the cord attachment site. This type of puncture is recommended since it is generally accomplished with the greatest ease and certainty. The drawback of this approach is that maternal and fetal blood would mix, potentially causing immunological sensitization and lowering test accuracy.

(b) Puncture of the umbilical cord attachment site on the posterior uterine wall - This approach has the potential to harm the fetus and is usually difficult to perform, but if the puncture can be done properly and only the umbilical vein is pierced, sensitization is uncommon. It's crucial to be cautious of contamination with maternal blood in this procedure, as it is with the others.

(c) Sensitization is more difficult with free loop puncture than with placental puncture, although it's not uncommon to be prepared to avoid the placenta. Because the cord is floating inside the amniotic fluid, the goal of this approach is to puncture it (usually the vena umbilicalis). Because the free loop will move quickly as the needle's tip comes into touch with it, the trick of this system is to puncture the cord as soon as the needle comes into contact with it. Fetal blood can be retrieved with 100% assurance if there is a distinct separation between the cord and, thus, the placenta. It is, nevertheless, difficult to carry out this task. Amniotic fluid emboli have also been reported, but these are much rarer [10]. 

When the placenta is anterior, cordocentesis is easier to accomplish. Because fetal parts are in the needle's route while the placenta is posterior, the procedure will be more difficult. In posterior placentas, the distance is higher. Cordocentesis has fallen out of favour since the polymer chain reaction technique for looking for infections in amniotic fluid was introduced, and it is now only utilised for late investigations (beyond 24 weeks) or intrauterine transfusion. Fetal loss occurs at a rate of roughly 2% of the time [11]. 

The most serious risk is still fetal pain during or after operation. If the gestational age is appropriate, an emergency caesarean delivery may be necessary, posing the risk of preterm birth or fetal/neonatal death. Fetal distress can be caused by cord rupture, spasm, tamponade from a cord hematoma, or excessive bleeding from the puncture site. Death that occurs as a result of a surgery might be linked to an already compromised fetal status or the procedure itself. Fetal loss is estimated to be between 0.9% and 4.9% for each treatment [12].

Potential risks of PUBS must be discussed during counselling, which may include bleeding from the puncture site (20%-30%), fetal bradycardia (5%-10%), pregnancy loss (1.3%) and transmission of hepatitis or HIV, but the current evidence is limited. Vertical transmission risks appear to be very low and related to maternal viral load [12].

## Conclusions

PUBS/cordocentesis is an advanced technique to be performed with caution in advanced settings for immediate caesarean delivery. Even though as advanced as it seems it is associated with significant risks and should only be used when it is absolutely indicated particularly in the setting of Fetal anemia for intrauterine transfusion as a fetus salvageable procedure when delivering the fetus at that gestational age is associated with more complications than performing PUBS. Pre-procedure preparation must include counselling the mother about the risks associated with the procedure and particularly about the risk of fetal loss associated with the procedure and a written Informed consent should be obtained. This review of literature has provided an overview of PUBS with emphasis on the uses and risks associated with PUBS over the years.
